# Seasonal Variation in Essential Oils Composition and the Biological and Pharmaceutical Protective Effects of* Mentha longifolia* Leaves Grown in Tunisia

**DOI:** 10.1155/2018/7856517

**Published:** 2018-12-09

**Authors:** Karama Zouari-Bouassida, Mohamed Trigui, Samar Makni, Lobna Jlaiel, Slim Tounsi

**Affiliations:** ^1^Laboratory of Biopesticides, Center of Biotechnology of Sfax, University of Sfax, P.O. Box 1177, 3018 Sfax, Tunisia; ^2^Analysis Department of the Center of Biotechnology of Sfax, University of Sfax, P.O. Box 1177, 3018 Sfax, Tunisia

## Abstract

This research assessed the seasonal variation of the chemical composition and antibacterial and anticholinesterase activities of essential oils extracted from* M. longifolia* leaves. The leaves organic fractions were also investigated for their biological activities and pharmacological functions. The essential oil highest yield was recorded in the spring season. Pulegone (26.92%), 1.8 cineole (21.3 %), and L-menthone (10.66 %) were determined as its major compounds in the winter season. In the spring oil, the main components were pulegone (38.2 %) and oleic and palmitic acids (23.79 % and 15. 26 %, respectively). Oxygenated monoterpenes were predominant in the two analyzed samples. The tested oils and organic extracts exhibited promising antibacterial effects against all of the tested bacterial strains. Thanks to its richness in phenolic and flavonoid compounds, the ethyl acetate fraction (Ml EtOAcF) displayed the most active DPPH scavenging ability (IC_50_ =12.64 *μ*g/ml) and an interesting *β*-carotene bleaching inhibition (IC_50_ =34.75 *μ*g/ml) making it a potential candidate for anti-inflammatory evaluation on rats. This evaluation evidenced that* M. longifolia *pretreated rats showed a marked decrease in paw oedema and inflammatory cells. Additionally, a remarkable acetylcholinesterase inhibitory activity of the Ml EtOAcF (IC_50_ = 12.3*μ*g/ml) and essential oils were also observed suggesting their neuroprotective property against Alzheimer's disease. Moreover, it was found that its activity level was season dependent. Our investigation, therefore, clearly revealed the medicinal characteristics of* M. longifolia* leave indicating their potential uses for natural remedies.

## 1. Introduction

Oxidative stress is one of the biological processes involved in the development of chronic diseases like inflammation, atherosclerosis, cancer, diabetes, and neurodegenerative diseases including Alzheimer [1]. Antioxidant molecules play a protective role against damage induced by free radicals and they are crucial in maintaining good human health [2]. Medicinal plants have been used in developing countries as alternative treatments to solve health problems. In recent years, scientists have focused remarkably on seeking natural antioxidant molecules to protect cells and tissues from biological damage induced by oxidative stress. Plant secondary metabolites, especially polyphenols, are naturally occurring compounds largely distributed in the plant extracts [3]. They have been reported to have potential protective roles against several human diseases and disorders, thanks to their antioxidant activity and enzyme-inhibitory capacity [4]. Previous studies highlighted that the anti-inflammatory efficiency has been attributed to these natural antioxidant compounds, suggesting them to be a new alternative against cellular injury induced by oxidative stress such as inflammation [5, 6].

Additionally, phenolic compound activities, acting as inhibitors of acetylcholinesterase (AChE), have also been revealed and were closely correlated with their structure and could be used as effective and safe Alzheimer's disease (AD) therapy [7]. AD results from decreasing cholinergic functions in the brain by rapid and excessive hydrolysis of acetylcholine [8]. Therefore, one of the most promising strategies for the treatment of this disease was to inhibit the AChE. Investigations of plants with an anticholinesterase action have therefore gained attention in this field. Many secondary plant metabolites have been shown to exert an anti-AChE activity [9] and therefore can be used as novel natural drugs reducing side effects and providing numerous beneficial effects on human health. Various studies are currently carried out in order to find adequate phytocompounds which can be used as activators of antioxidative defence enzyme systems to delay or suppress the cellular damage in biological systems [10, 11].

Thus, natural products from plants having antioxidant capacities might be a promising alternative to generate new multitargeting bioactive compounds.

The Tunisian flora is characterized by a large diversity of aromatic and medicinal plants.* M. longifolia* L is a perennial and aromatic herb which belongs to* Lamiaceae* and commonly known as “hbak” in Tunisia. Total decoction of* M. longifolia *L. has been traditionally used for the treatment of many respiratory disorders such as cold, bronchitis, and sinusitis as well as gastrointestinal disorders such as stomach problems, diarrhea, and abdominal pain [12]. The* M. longifolia* essential oil has also been scientifically known for its analgesic, antimicrobial, antioxidant, and antiplatelet properties [13–15].

Previous studies on the chemical composition and medicinal properties of* M. longifolia* have mainly focused on its crude extract and essential oil, but only few reports have dealt with the organic fractions of the plant and searched for their anti-inflammatory and anticholinesterase activities as well. The present study aimed mainly to (i) assess the essential oil content and composition of* M. longifolia* with regard to time of its leaves collection; (ii) investigate the influence of the seasonal variation on the antioxidant, antibacterial, anticholinesterase capacities of* M. longifolia *leave essential oils; (iii) explore the* in vivo* anti-inflammatory efficiency of the most active fraction, Ml EtOAcF, with reference to its phenolic compounds richness and antioxidant and antimicrobial effects.

## 2. Materials and Methods

### 2.1. Essential Oil Extraction and Chemical Analysis

*M. longifolia* fresh leaves were harvested from Jelma Sidi Bouzid (Tunisia, latitude 35.36° and longitude 9.38°). Plant samples were collected twice: during spring (April-May) and winter at the full ripening (October-November). The plant specimen was authenticated by Professor Mohamed Chaieb, and a voucher sample was kept at the Biopesticides Laboratory Herbarium of the Centre of Biotechnology of Sfax under the code number LBPes 04.

The essential analysed oils in this study were extracted by hydrodistillation of* M. longifolia* fresh leaves (100g) for 3 h using a Clevenger-type apparatus. The essential oils were performed in triplicate for each oil sample. The oil was extracted with dichloromethane (3×50 ml) and dried with anhydrous Na_2_SO_4_. For yield determination, the solvent was evaporated using a rotavapory vacuum evaporator (BUCHI R-200), the yields were calculated according to dry weight of the plant material. The resulting samples were transferred to opaque glass and stored at 4°C during the experimental period. The* M. longifolia* essential oil was diluted in* n*-Hexane for gas chromatography-mass spectrometry analysis (GC-MS).

### 2.2. Gas Chromatography-Mass Spectrometry (GC-MS) Analysis

GC-MS analysis of the* M. longifolia* essential oil was performed with an Agilent 6890N Network GC system (Agilent Technologies). The system was equipped with an HP-5 MS column having 30 m × 0.25 mm i.d. × 0.25*μ*m film as dimensions. The used system was coupled to a mass selective detector and the carrier gas was Helium. The GC oven temperature started at 40°C and was held at 100°C for 1 min and then programmed to rise from 100 to 280°C at a rate of 5°C/min. The identification of essential oil constituents was performed by comparing their kovats index and mass spectra with those of the authentic standards stored on the Wiley Registry of Mass Spectral Data 7th edition (Agilent Technologies, Inc.) and National Institute of Standards and Technology 05 MS (NIST) library data.

### 2.3. Preparation of* M. longifolia* Leaf Extracts

The* M. longifolia *powdered leaves (from winter crops) were crumbled into small parts using a blender and were macerated three times in ethanol-water (8:2, v/v). The resulting extract was filtered and ethanol was evaporated and lyophilized to yield the hydroalcoholic crude extract (Ml EtOH-H_2_OE). The dried hydroalcoholic crude extract (23g) was sequentially partitioned with n-hexane (3 × 350 ml) and ethyl acetate (3 × 350 ml). The filtered solution was evaporated at reduced pressure (Rotary Evaporator Buchi R-200, Switzerland) and the remaining aqueous layer was lyophilized to give the water fraction. The resulting three fractions were evaporated under vacuum to dryness to give the hexane (Ml HexF), the ethyl acetate (Ml EtOAcF), and water (Ml WF) fractions. The stock solutions were kept at 4°C in the dark until tested and analyzed.

### 2.4. Determination of Total Phenols (TPC)

The TPC in extracts was determined according to the Folin-Ciocalteu procedure with slight modifications [16]. A calibration curve was constructed using gallic acid and the results were expressed as mg gallic acid equivalent (GAE)/g. Tests were performed in triplicate for each extract.

### 2.5. Determination of Total Flavonoids (TF)

The assay was performed spectrophotometrically following the procedure described by Quettier-Deleu et al. [17] based on the formation of a complex flavonoid-aluminium, having a maximum absorption at 430 nm. The flavonoid content was expressed in mg of quercetin equivalent per gram of dry plant extract (mg QE/g).

### 2.6. *In Vitro* Antioxidant Assay

#### 2.6.1. DPPH Antiradical Activity

The capacity of* M. longifolia* leave extracts (essential oil; hydroethanolic extract and its fractions) to scavenge the free-radical 2, 2-diphenyl-1-picrylhydrazyl (DPPH) was determined according to the method described by Trigui et al. [18] with slight modifications. In Brief, a dosage of 50 *μ*l of the extract assayed at different concentrations was added to 2 ml of a DPPH solution (0.04 g/l in methanol). The mixtures were incubated for 30 min in the dark at room temperature.

The DPPH radical reduction was evaluated spectrophotometrically by continuous monitoring of the absorption decrease at 517 nm. Ascorbic acid was used as a positive control. The DPPH scavenging effect percentage was calculated using the following formulae:

(1) $$\begin{array}{c} \left[\;\frac{1-Aextract}{ADPPH}\;\right]\times 100 \end{array}$$

where Aextract is the absorbance of the solution when an extract is added and ADPPH is the DPPH solution absorbance. The IC_50_ was then calculated (the fraction concentration that inhibits 50% of free radicals).

#### 2.6.2. *β*-Carotene Bleaching Assay

The ability of the* M. longifolia* leave extracts to prevent the bleaching of *β*-carotene was assessed as previously described by Trigui et al. [18]. A solution of *β*-carotene/ linoleic acid was prepared by dissolving 0.2mg of *β*-carotene, 20 *μ*l of linoleic acid, and 200mg of Tween 40 in 1ml of chloroform. After chloroform evaporation under reduced pressure at 40°C, 50ml of oxygen bubbled water was slowly added to the residue and the resulting mixture was vigorously shaken. Aliquots (5ml) of the obtained emulsion was added to a tube containing 500 *μ*l of each extract and kept in a water bath at 50°C for 120 min before measuring the absorbance at 470 nm. The butylated hydroxytoluene (BHT) was used as the reference. The antioxidant activity is expressed in terms of percent inhibition using the following formula:

(2) $$\begin{array}{c} Inhibition\%=\left[\;\frac{A\beta -\text{carotene after 2h assay}}{A\text{ initial }\beta -\text{carotene}}\;\right]\times 100 \end{array}$$

where A*β*-carotene after 2 h assay and A initial *β*-carotene are the absorbance of *β*-carotene after 2 h assay and the absorbance of *β*-carotene at the beginning of the experiments, respectively. The necessary antioxidant concentration to reduce 50% of the absorbance (IC50) was determined. Tests were performed in triplicate for each extract.

### 2.7. *In Vitro* Antibacterial Activity

Different microorganisms, including Gram-positive bacteria (*Bacillus subtilis* ATCC* 6633, Bacillus cereus* ATCC14579,* Staphylococcus aureus* ATCC 25923,* Staphylococcus epidermidis* ATCC 12228,* Enterococcus faecalis* ATCC 29212,* Micrococcus luteus* ATCC 1880, and* Listeria monocytogenes* 2132) and Gram-negative bacteria (*Salmonella enteritidis* and* Klebsiella pneumoniae* ATCC 10031) were used for testing the antibacterial activities of* M. longifolia* essential oils and fractions. Mueller-Hinton agar (MH) (Oxoid Ltd., UK) was used for the bacterial strains cultivation for 24 hours at the appropriated temperature. The agar-well diffusion assay was employed for this purpose [19]. The minimum inhibitory concentrations (MICs) were determined using the microdilution bioassay in 96-well microplates according to Eloff et al. [20] with minor modifications. Gentamicin was used as positive control. All tests were carried out in triplicates.

### 2.8. *In Vitro* Anticholinesterase Activity

The acetylcholinesterase (AChE) inhibitory activity was carried out using the spectrophotometric method developed by Mata et al. [21] using electric eel acetylcholinesterase (EC 3.1.1.7, type VS). The absorbance of the mixture was measured at 405 nm. Acetylthiocholine iodide (AChI) was employed as substrate of the reaction and 5, 5′-Dithio-bis-2-nitrobenzoic acid (DTNB) was used for the measurement of the anticholinesterase activity. The galanthamine·HBr was used as positive control. The obtained results were expressed as IC_50_ values calculated as the extracts concentration that can produce 50% inhibition activity again cholinesterase.

### 2.9. *In Vivo* Anti-Inflammatory Test

#### 2.9.1. Animals

Wistar rats with body masses around 175 g were obtained from the Veterinary Research Institute (Sfax, Tunisia). The animals were caged under environmental conditions of humidity, temperature and dark/light cycle with free access to diet and water. The animal study was performed in accordance with the European Community guidelines (EEC directive of 1986; 86/609/EEC) for the care and use of laboratory animals in scientific research and approved by the Medical Institutional Animal Ethics Committee (Directive 2001-2133) issued by the University of Sfax Tunisia.

#### 2.9.2. Acute Toxicity Study

The acute toxicity study was performed according to the World Health Organization recommendations (2000) with some modifications. Healthy rats (175g) were randomly divided into five groups (n=10). The control group (group I) received only a solution of Tween-80 (3%), while groups II, III, IV and V were treated with Ml EtOAcF at doses of 100, 200, 400, and 800 mg/kg body weight, respectively. Following the fasting period, the graded doses of Ml EtOAcF dissolved in Tween-80 (3%), were administered orally by gavage. The animals were maintained on standard animal diet and water. All animals were daily observed for behavioral pattern, body weight, and physical appearance changes and checked for mortality during the 2-week observation period.

#### 2.9.3. Anti-Inflammatory Test: Carrageenan Induced Paw Oedema

The rats were split into four groups (n=10) pretreated, respectively, with 1 ml/kg of sterile saline solution (control group), carrageenan 1% (control group), 10 mg/kg BW of indomethacin as a standard group, and 200mg/kg of Ml EtOAcF [22]. Paw oedema was induced [23] by subcutaneous injection of 50*μ*l of 1% w/v carrageenan solution (type IV Sigma Chemical Company, USA) into the foot pad of the right hind paw after one hour of intraperitoneal injection of plant extract and drug. The paw oedema thickness was measured at hourly interval (from 0 hour up to 5 hours) using a caliper and compared with the control group [24]. The drug efficiency and carrageenan induced oedema were estimated using the following formula:

(3) $$\begin{array}{c} \%\text{ inhibition oedema}=\frac{T-T0}{T}\times 100 \end{array}$$

where T represents the paw thickness in the control group and T0 the paw oedema thickness in the test compound treated group.

Five hours after the carrageenan administration, the animals were anesthetized and the biopsies from the subplantar muscles of all groups of rats were collected for the paws histological examination.

### 2.10. Statistical Analysis

Data were expressed as mean values ± standard deviation (SD). A statistical significance comparison between groups was accomplished using the SPSS version 20.

The mean differences between the different groups were assessed by Duncan and Tukey's post hoc tests and compared using one way analysis of variance (ANOVA). Differences were considered significant at* P* < 0.05.

## 3. Results and Discussion

### 3.1. Seasonal Variation in* M. longifolia* Essential Oil

The oil yield-revealed data are shown in Table 1. The largest amount of oil was found during the winter season corresponding to the flowering stage (2.5%) whereas the* M. longifolia* oil content decreased significantly (*P* < 0.05) in the spring season to reach 0.5%. The results revealed that essential oil accumulation in* M. longifolia* appears to be metabolically regulated during vegetative and flowering stages of crop growth. Such results were expected as the flowering stage is well known to be the most suitable for essential oil extraction compared to vegetative stage [25] and were in agreement with other research findings reporting a maximum essential oil yield during flowering stage [26]. It was also shown by Hussain et al. [27] that many tested* Mentha* species revealed higher essential oil yield when the plants were in full bloom than when they reached the end of their growth cycle. The climatic factors variability such as temperature, total duration of exposure to sun, and precipitation could directly influence the oil yield during the two harvesting seasons [28, 29]. It was reported that the temperature influenced the oil yield of* O. basilicum* with the highest yields in the winter season [30].

The mean percentage composition of total identified compounds of essential oils collected through the two seasons and used in bioassays are presented in Table 2. A total of eighteen and sixteen compounds, accounting for 89.91 and 97.95 % of the total content, were identified in the essential oils obtained from winter and spring leaves, respectively. The oxygenated monoterpenes fraction dominated the two essential oils samples.

The content of oxygenated monoterpenes in the essential oils was higher during the winter (81.19%) and lower during the spring (53.48%). Apart from the similarity in the major compound, the oils collected in winter and spring showed notably quantitative differences. In the winter crops, the prominent components were pulegone (26.92%), L-menthone (10.66 %), and 1,8 cineole (21.3 %). However, the major constituents in the spring oil were found to be pulegone (38.2 %) and fatty acids. This oil harbors also menthone (4.68%), menthol (4.29%), and 1,8 cineole (2.37%) but with less quantities. Fatty acids, not detectable in the winter oil, were represented mainly by oleic acid (23.79%) and palmitic acid (15.26%) and to a lesser extent by linoleic acid (3.27%) in the postflowering stage essential oil. Moreover, the tested* M. longifolia* essential oils contained significant amounts of several minor constituents, as seen in Table 2, including sesquiterpene hydrocarbons, oxygenated sesquiterpenes, and esters.

The variation in the composition of the* M. longifolia* essential oils investigated in this study was quantitatively significant (*P* < 0.05). Most fluctuations in the essential oil components includes oxygenated monoterpenes, namely, pulegone (26.92-38.2%), 1, 8 cineole (2.37-21.3 %), L- Menthone (4.68-10.66 %), *β* pinene (0.24-1.22), *α* pinene (0.12-0.93 %), cis iso-pulegone (0-0.86 %), and piperitenone (1.18-9.62 %) from spring and winter, respectively. The yield of most of the compounds was higher in the flowering stage (winter), while pulegone was found to be high in the spring plant collection.

Our findings are in line with those of Gazim et al. [31], who stated essential oil composition variation obtained from* Tetradenia riparia* (*Lamiaceae*) leaves at different seasons. The oxygenated monoterpenes and oxygenated sesquiterpenes contents were high during the winter and low during the spring-summer.

The seasonal variations in content and chemical composition of* M. longifolia* essential oil across countries have been attributed to environmental factors (climate/weather, soil/nutrition) that can influence the regulation of the essential oil biosynthesis [32]. Just like our results, previous investigations have demonstrated that the harvesting season can alter the chemical composition of the essential oils of* M. longifolia* [30].

Thus, based on these results and previous works we can conclude that the ingredients of* M. longifolia* essential oil revealed a variation in its constituents depending on geographical and environmental factors and harvest time. Indeed, the adaptive metabolism of the plant influences the quality, the quantity and the chemical composition of the plant essential oil and could probably contribute to create a unique and specific chemical composition.

Several previous reports from the literature show the qualitative and the quantitative analyses of* Mentha longifolia* essential oil from different countries [33–35].

### 3.2. Total Phenolics (TPC) and Flavonoids (TFC)

The quantitative estimation of TPC of the* M. longifolia* leave extracts (Table 1) showed that they are upper than their TFC amounts. The content of these secondary metabolites depends on the type of solvent used for the extraction with varying polarity.

The Ml EtAOcF was found to contain the highest amount of phenolics (99.72 ± 1.26 mg GAE/g) compared to Ml EtOH-H_2_O extract (59.25 ± 0.38 mg GAE/g) and Ml WF (45.25 ± 0.87 mg GAE/g). The Ml EtAOcF also contained the highest flavonoid concentration (20.85 ± 0.94 mg QE/g), 2-fold more than Ml WF with 10.75 ± 0.47 (Table 1). The higher TPC and TFC values obtained with the ethyl acetate can be attributed to its good phenolic solubility and the high extraction capacity [36]. These metabolites showed differences in their concentrations in terms of solvents polarities and therefore their solubility which depends on their structures and polymerization degree [37].

### 3.3. Anticholinesterase Inhibition Assay

The acetylcholinesterase inhibitors are one of the most effective approaches to treat the cognitive symptoms of the Alzheimer's disease (AD).  Table 3 shows the antiacetylcholinesterase capacity of essential oils and organic extracts of* M. longifolia*, compared with those of galantamine used as a standard inhibitor.

Based on IC_50_ values, all the extracts exhibited acetylcholinesterase inhibitory ability in the following order: Ml EtOAcF > Ml EtOH-H_2_OE > Ml WF > Ml HexF. The uppermost inhibitory activity was recorded with the Ml EtOAcF (IC_50_ = 12.30 *μ*g/mL). Besides, it is clearly shown that AChE inhibitory activity of* M. longifolia* essential oils varied significantly along their harvesting time.  Table 3 shows that the essential oil extracted at the flowering stage (winter crops) exhibited the greatest inhibitory effect against AChE with an IC_50_ of 21.9*μ*g/ml. Previous studies reported that the* M. longifolia* leaves essential oils have an acetylcholinesterase inhibitory capacity [38]. However, so far, and to the authors best knowledge, this is the first attempt that explores the seasonal variation of the AChE inhibitory capacity of* M. longifolia* essential oils.

Monoterpenes, reported in* M. longifolia *essential oils, are known for their biological activities and beneficial effects [39]. Numerous essential oils and their isolated monoterpenes have been investigated for their effects on AChE. It was proven that purified 1.8-cineole, a monoterpene one of the major compounds of winter* M. longifolia *essential oil, exhibited a potent inhibitory capacity on AChE activity and showed synergistic effect with other constituents [40]. It was reported by Mata et al. [21] that the essential oils of the two* Mentha *species,* M. pulegium* and* M. spicata*, exhibited only moderate AChE inhibition with an IC_50_ of 324 and 357 *μ*g/ml, respectively. The main components of these oils were pulegone and carvone. These compounds are reported as moderate inhibitors of AChE [41]. According to previous observed data, the AChE inhibition in our case by* M. longifolia *essential oil (IC_50_ = 21.9 *μ*g/ml) led to the suggestion that the activity is probably due to the synergistic effects of several components major and minor ones mainly monoterpenoids.

On the other hand, the Ml EtOAcF was found to be more effective in the AChE inhibition than essential oils. Differently, Lopez et al. [42] reported an insignificant inhibition of organic extracts of* M. longifolia* on this enzyme.

Plants have been traditionally used to enhance cognitive functions and alleviate other symptoms nowadays associated with Alzheimer's disease [43]. Most of the drugs used in Alzheimer therapy are formed by an enzyme inhibitor, e.g., galanthamine, isolated from the snowdrop extract [44]. There has been a lot of research on the biological effect of plants traditionally used as acetylcholinesterase inhibitors* in vitro *and also as memory enhancers* in vivo *[45, 46]. The great inhibitory effect of Ml EtOAcF made us think that this fraction contains neuroprotective phytochemical molecules like phenolic active compounds which could be responsible for the plant potent anti-AChE activity. The AChE inhibition efficacy of Ml EtOAcF observed in this work comes probably from its richness in various effective chemical compounds present in the extract such as phenolic acids and flavonoids, proven to have an inhibitory ability against key enzymes involved in the cholinergic nervous system [47]. The bioactive compounds can act synergistically or individually to induce an AChE inhibitory activity. Further analyses have to be performed to delineate rationally the Ml EtOAcF active compound(s).

### 3.4. Evaluation of Antioxidant Activity: DPPH and *β* Carotene Tests

The effect of the different* M. longifolia *organic extracts and essential oils on DPPH radical scavenging capacity was evaluated by the determination of the IC_50_ values and then compared to the ascorbic acid used as positive control. Based on the IC_50_ values, the best activities were recorded with Ml EtOAcF (IC_50_= 12.64 *μ*g/ml), followed by Ml EtOH-H_2_OE (IC_50_ = 16.23 *μ*g/ml) and then by Ml WF (IC_50_ = 47.71± 0.43*μ*g/ml). The Ml HexF showed a poor radical scavenging activity with an IC_50_ value more than 1000 *μ*g/ml (Table 1). The Ml EtOAcF showed an interesting free-radical scavenging. Ascorbic acid showed a potent antioxidant activity with an IC_50_ value of 3.5 *μ*g/ml (Table 1).

Both samples of* M. longifolia *essential oil showed a weak DPPH radical scavenging activity with an IC_50_ value more than 100 *μ*g/ml when compared to the organic extracts. Our results are in accordance with the findings of Gulluce et al. [48] who reported that* M. longifolia *methanol extract (obtained at the flowering stage) was more effective in scavenging DPPH free radical than the essential oil. The antiradical efficiency due to a hydrogen atom transferring reaction showed a positive correlation with phenolics content (R^2^ = 0.987) and therefore their implication in* M. longifolia *antiradical activity.

A wide variation in the antioxidant activity of* M. longifolia *was observed in the literature. Mkaddem et al. [49] showed a moderate IC_50_ value of essential oil (> 8000 mg/ml) assayed by DPPH. It was also found that the IC_50_ value of the methanol extract of Turkish* M. longifolia* was in the range of 57.4 *μ*g/ ml [48]. In addition, a lower IC_50_ of methanol extract in the range of 20 *μ*g/ml was obtained by Hajlaoui et al. [50].

All* M. longifolia *organic extracts inhibited the *β*-carotene oxidation except for essential oils. The Ml EtOAcF exhibited a strong activity with IC_50_ values of 34.75 *μ*g/ml compared to those of the essential oils (IC_50_ > 100 *μ*g/ml). The inhibition of lipid peroxidation by addition of* M. longifolia *organic extracts could be used to improve the quality and stability of food products. These extracts are able to quenching peroxide radicals and to terminate the peroxidation chain reaction.

These results suggest that* M. longifolia* leaves are promising sources of various bioactive compounds for human health. The results obtained for antioxidant properties in this work are in agreement with earlier results of other authors [34, 48].

### 3.5. Antibacterial Activity

The inhibitory spectra of the essential oils seasonally extracted from* M. longifolia* and its organic extracts were evaluated against nine pathogenic bacteria by the presence or absence of inhibition zones followed by MIC and MBC values measurement (Table 4).

The results indicated that* Bacillus subtilis, Bacillus cereus, Staphylococcus aureus, and Enterococcus faecalis* were the most sensitive microorganisms to the essential oils. They also exhibited the lowest MIC values varying between 0.078 and 0.156mg/ml, 0.156 and 0.312 mg/ml, 0.312 and 0.625 mg/ml, and 0.078 and 0.625mg/ml, respectively. As shown in Table 4, the* M. longifolia* harvest time affected significantly the inhibitory power of the essential oils (*P* < 0.05). It was found that the activity against* Bacillus subtilis, Bacillus cereus, Staphylococcus aureus and Enterococcus faecalis* was highest in winter. The potent antibacterial activity of* M. longifolia* essential oil sample from winter crops could be attributed to its richness in oxygenated monoterpenes (81.19%). It was reported that an essential oil containing monoterpenoid class compounds showed remarkable antimicrobial activities [51, 52].

Pulegone, 1.8-cineole, and L-menthone were proven to have a potential antibacterial activity. Therefore, the winter essential oil efficiency may be especially related to its richness in such compounds. Pulegone was found to have an antibacterial activity through dissipating the pH gradient and altering the cell membrane potential [53]. Li et al. [54] reported that 1.8-cineole exhibits strong antibacterial properties that can be attributed to its ability to directly produce alterations on the structure of* E. coli*,* S. enteritidis* and* S. aureus*. Simsek et al. [55] revealed that 1.8-cineole extracted from* Eucalyptus* essential oils could improve the antimicrobial effects of other antiseptics against some microorganisms such as* S. aureus*,* E. coli*, and* E. faecalis.* These composites may be able to inhibit synergistically or individually the growth of microorganisms and fight against bacterial infections.

Our results also showed that essential oil samples exhibited high antibacterial activity against all Gram-positive bacteria tested. Among the Gram-negative ones,* Salmonella enteritidis* showed a lower sensitivity than* Klebsiella pneumonia *(Table 4). Furthermore, this activity within these strains was not affected by seasonal variation. According to previous reports, Gram-negative bacteria appeared to be less effective in the action of various plant essential oils. This resistance against Gram-negative ones might be related to their highly complex outer membrane which protects them [56]. The Gram-positive bacteria cell membrane may facilitate penetration of the hydrophobic compounds [57]. However, the way the components of essential oil interact to inhibit bacterial growth could be a potential future perspective.

This study showed that the harvest period influences the percentage of chemical constituents of* M. longifolia* essential oil and that the observed essential oil antibacterial characteristics may be related to the oil chemical composition variation.

Among the tested extracts, only Ml EtOAcF and Ml EtOH-H_2_OE exhibited an antibacterial activity. Ml HexF and Ml WF remained inactive in the range of the used concentration (2.5 mg/wells). Active extracts showed a potent antimicrobial activity against both Gram-positive and Gram-negative bacteria. The inhibition zone diameters and MIC values were in the range of 17-27 mm and 0.039-1.25 mg/ml for the Ml EtOAcF and 12-20 mm and 0.078-2.5 mg/ml for the EtOH-H2OE. The Ml EtOAcF was the most active fraction with the lowest MBC values ranging from 0.0.78 to 1.25 mg/ml, respectively (Table 4).

Based on these results, it is possible to conclude that Ml EtOAcF has a stronger and broad spectrum of antibacterial activity compared to the essential oil and EtOH-H_2_OE. The observed differences in the inhibition zones within pathogenic bacteria could probably be due to cell membrane permeability or other genetic factors.

Furthermore, Ml EtOAcF and Ml EtOH-H_2_OE displayed a potent inhibition against* Bacillus *sp*., L. monocytogenes*, and* S. enteritidis* with an MIC that ranged from 39 to 156 *μ*g/ml. Infections caused by these bacteria, especially those with multidrugs resistance, are among the most difficult to treat with conventional antibiotics. In the current study, the growth of* B. subtilis* was remarkably inhibited by the Ml EtOAcF (IZ = 27 mm; MIC = 0.039 mg/ml; and MBC = 0.078 mg/ml). The Ml EtOAcF remarkable antibacterial activity could be mainly due to its richness in composites that are well known by their antibacterial properties such as polyphenols and flavonoids found abundantly in this fraction [58]. Previous studies reported that these compounds are therefore able to minimize problems of drug resistance [59]. Earlier reports demonstrated that an interaction between signal transduction pathways and cell receptors and chemicals like polyphenols leads to the interruption of microorganisms growth and results in the cell death [60]. These results revealed that Ml EtOAcF can be used to manage bacterial resistance and protect foods against multiple pathogenic bacteria.

According to its strong antibacterial and antioxidant activities, the Ml EtOAcF was chosen to be evaluated for its anti-inflammatory efficiency in rats' models.

### 3.6. Acute Toxicity Study

The Ml EtOAcF up to the dose of 800 mg/kg BW did not produce any signs of adverse reactions and no changes on the behavior of the treated animals up to 14 days following the extract administration. The treated animals did not display any abnormal signs such as food and water intake, convulsions, salivation, or diarrhea. No deaths or weight losses were recorded during the study. Therefore, the Ml EtOAcF at 200 mg/Kg BW was used in the* in vivo* investigation of the anti-inflammatory activity.

### 3.7. Anti-Inflammatory Activity

**Figure 1:**
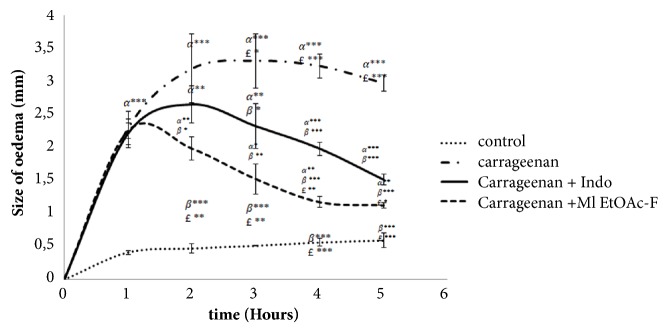
Effect of Ml EtOcF and indomethacin on paw oedema induced by carrageenan. Values represent mean ± SD (*n* = 6) in each group. *∗P* < 0.05, *∗∗ P* < 0.01, and *∗∗∗ P* < 0.001. *α*: compared to control; *β*: compared to Carr; £: compared to Carr + Indo. Control: saline solution, Carr: carrageenan, Carr + Ml EtOcF: carrageenan+* M. longifolia *ethyl acetate fraction; Carr + Indo: carrageenan + indomethacin.

In our study, subcutaneous administration of carrageenan induced an increase in paw size in rats due to oedema, thus revealing an acute paw inflammation.  Figure 1 shows the paw thickness in the different studied groups. The Ml EtOAcF and indomethacin significantly reduced carrageenan induced paw oedema in rats (*P *< 0.001) five hours after the induction of inflammation when compared to the control group.

Our findings revealed an inhibition of 62.29% of the paw oedema using the Ml EtOAcF compared to only 49.15% inhibition using the standard drug indomethacin after five hours (*P* < 0.01). The anti-inflammatory effect of 200 mg/kg BW of Ml EtOAcF was comparable to that of the standard drug indomethacin (Figure 1).

**Figure 2:**
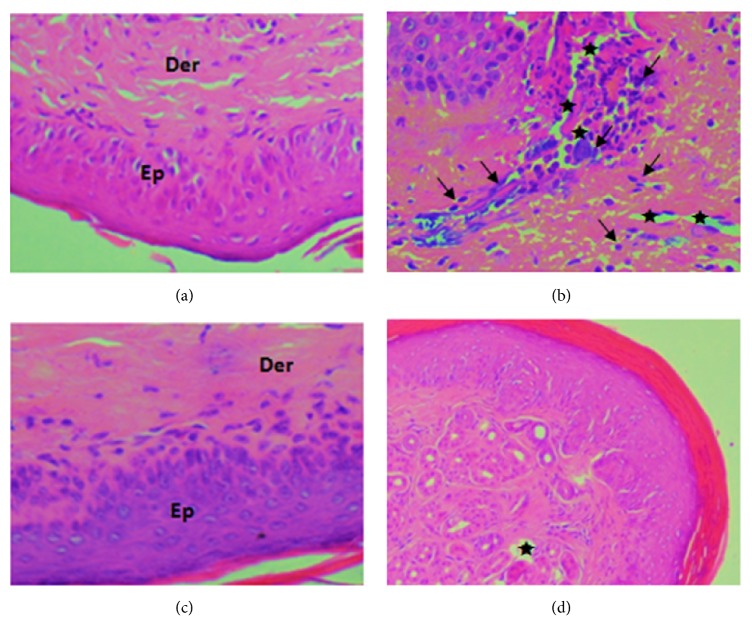
Histopathological slides tissues of oedema paws in experimental groups of rats: (a) Saline group; (b) Carr group; (c) Carr + Ml EtOcF group (200 mg BW); (d) Carr + Indo group. Controls (a), rats treated with carrageenan (b), the combination of carrageenan and indomethacin (c), and treated rats with the combination of carrageenan and Ml EtOcF (d). Ep: epidermis; Der: dermis. Oedema ★. Inflammatory cell *↗*.

The paw oedema tissues from all the groups were histologically examined (Figure 2). The control group showed normal tissue whereas the carrageenan inflamed group displayed an intense oedema, characterized by conjunctive tissue with a substantial number of inflammatory cells infiltration. The carrageenan groups pretreated with indomethacin (10 mg/kg) or Ml EtOAcF (200mg/kg) exhibited a significant decrease in oedema as well as reduction in the inflammatory cells (Figure 2).

The development of oedema inflammation is a biphasic event: the first phase is caused by the release of histamine, leukotrienes, bradykinin, and cyclooxygenases in the first hour of the injection of carrageenan, and the second phase is linked to an increased generation of prostaglandins in the inflammatory tissue [61]. In the present study, Ml EtOAcF significantly improved the paw oedema induced by carrageenan after 5 hours. This result suggests that the Ml EtOAcF anti-inflammatory efficacy may be due to the inhibition of cyclooxygenase synthesis. Such an effect is similar to that produced by nonsteroidal anti-inflammatory drugs such as indomethacin [62]. However, the exact mechanism of how Ml EtOAcF inhibits cyclooxygenase biosynthesis could be part of our future perspectives.

## 4. Conclusion

The study highlighted the effect of the harvesting time on the essential oils composition and biological activities of* M. longifolia*. The essential oils extracted from the fresh leaves were characterized by their richness in oxygenated monoterpenes. Significant variations of the essential oils composition were shown depending on the harvesting time.* M. longifolia* essential oils have a remarkable antibacterial activity and an effective inhibitory effect against the enzyme related to Alzheimer's (AChE). However, the levels of these biological activities vary according to the harvest time. In fact, the interesting antibacterial and AChE inhibitory activities were observed in the winter crops, corresponding to the flowering stage. These results may be useful in choosing the optimal collection time for the production of the plant active agents with high medicinal value. The present study also revealed that the Ml EtOAcF obtained from winter leaves has a significant anti-inflammatory capacity and the uppermost anticholinesterase activity. The richness of the extract in various chemicals such as phenolic compounds could clearly contribute to its medicinal properties. Further studies are still recommended to accurately identify the bioactive molecules in this extract for the exploitation of this plant in various pharmaceutical fields and the promotion of the desired biological activities.

## Figures and Tables

**Table 1:** Yields extracts, amounts of total flavonoids, total phenolic compounds, and determined IC_50_ values of the DPPH free radical scavenging assay of *M. longifolia *leave extracts and essential oil. Ascorbic acid was used as positive control.

**Extracts**	**Yield (**%**)**	**TPC** **mg GAE/g**	**TF** **mg QE/g**	**DPPH** **IC**_**50**_** (**μ**g/ml)**	β** carotene** **IC**_**50**_** (**μ**g/ml)**
Ml EtOH-H_2_OE	12.46	59.25±0.38	18.23±0.35	16.23 ± 1.60	66.75± 0.57
Ml HexF	7.35	nd	nd	> 1000	>1000
Ml EtAOcF	2.5	99.72±1.26	20.85 ± 0.94	12.64± 0.21	34.75 ± 0.76
Ml WF	2.61	45.25 ± 0.87	10.75 ± 0.47	47.71± 0.43	56.31± 0.67
EO_**s**_-EO_**w**_	0.5-2.5	-	-	> 100	> 100
Ascorbic acid	-	-	-	3.50	-

TPC: total phenolic content; TF: flavonoid content.

mg GAE /g: mg of gallic acid equivalent per g of dry plant extract.

mg QE/g: mg of quercetin equivalent per g of dry plant extract.

IC_50_ (*μ*g/ml): the IC_50_ values corresponding to the amount of extract required to scavenge 50 % of radicals present in the reaction mixture.

nd: not detected.

EO_**w**_: essential oil extracted at winter season; EO_**s**_: spring essential oil.

**Table 2:** Seasonal variation in content and chemical composition of essential oils from leaves of *M. longifolia*.

**Component** ^**A**^	**Rt (min)** ^**B**^	**KI** ^**C**^	%** Area**	
			**Spring**	**Winter**
***Monoterpene hydrocarbons***				
*α*-pinene	8.557	941	0.12±0.008^b^	0.93±0.02^a^
camphene	9.764	953	0.18±0.03^b^	0.41±0.008^a^
*β*-pinene	9.827	978	0.24±0.02^b^	1.22±0.05^a^
***Oxygenated monoterpenes***				
1,8 cineole	11.492	1030	2.37±0.16^b^	21.3±0.24^a^
L-menthone	15.206	1152	4.68±0.2^b^	10.66±0.1^a^
Iso-menthone	15.429	1163	0.16±0.008^b^	8.69±0.12^a^
Borneol	15.503	1166	2.6±0.02^a^	1.87±0.04^b^
Menthol	15.801	1171	4.29±0.2^a^	0^b^
Cis-isopulegone	17.960	1175	0^b^	0.86±0.04^a^
Pulegone	18.349	1237	38.2±0.28^a^	26.92±0.53^b^
Piperitone	19.539	1251	0^b^	0.82±0.1^a^
Piperitenone	21.324	1340	1.18±0.12^b^	9.62±0.2^a^
cis-Jasmone	22.406	1400	0^b^	0.45±0.02^a^
***Sesquiterpene hydrocarbons***				
trans-Caryophyllene	22.949	1419	0.87±0.04^b^	2.7±0.1^a^
D-germacrene	24.046	1482	0.34±0.008^a^	0^b^
gamma.-Cadinene	25.106	1514	0^b^	0.52±0.08^a^
d-Cadinene	27.607	1523	0.4±0.006^a^	0^b^
delta.-Selinene	27.857	1578	0^b^	0.30±0^a^
***Oxygenated sesquiterpenes***				
alpha.-Humulene	23.659	1454	0^b^	0.2±0^a^
alpha.-Cadinol	28.196	1654	0^b^	2.81±0.05^a^
***Esters***				
Geranyl tiglate	29.260	1685	0^b^	0.24±0.008^a^
***Fatty Acids***				
Palmitic acid	34.729	1927	15.26±0.2^a^	0^b^
Oleic acid	38.002	2128	23.79±0.5^a^	0^b^
Linoleic acid	38.317	2173	3.27±0.01^a^	0^b^
Total compound			97.95	89.91

^A^Compounds listed in order of elution from a HP-5 MS column.

^B^Retention time (as minutes).

^C^K.I. Kovats Index on HP-5MS column in reference to n-alkanes.

Values in the same line with different subscript (a > b) are significantly different within season (*P *< 0.05).

**Table 3:** Acetylcholinesterase inhibition capacity represented by IC_50_ (*μ*g/ml) of essential oils and organics extracts of *Mentha longifolia*.

**Extracts**	**Percentage of inhibition IC**_**50**_ ** (**μ**g/ml)**
Ml HexF	934± 42.3^c^
Ml EtOH-H_2_OE	54.25 ± 1.10^ab^
Ml EtOAc-F	12.3± 0.71^a^
Ml WF	85.68 ± 3.4^b^
EO_W_	21.9± 1.14^a^
EO_S_	49.3± 1.56^ab^
Galanthamine^*∗*^	19.90 ± 1.48^a^

Averages ± SD were obtained from three different experiments.

^*∗*^Standard drug.

EOw: essential oil extracted at winter season; EOs: spring essential oil.

Values in the same line with different subscript (a > b > c) are significantly different within season (*P* <0.05).

**(a):** 

**Bacterial strains**	**Extracts**				
	**Ml EtOcF**	**Ml EtOH-H**_**2**_ **OE**	**E** **O**_W_	**E** **O**_**S**_	**Genta** ^**∗**^
**Diameter of inhibition zone (mm)**					

**Gram ** ^+^					

*B. subtilis * ATCC 6633	27±0.1^a^	14±0.2^c^	26.3±0.0^a^	11±0.5^d^	20±0.2^b^

*B. cereus * ATCC 14579	17±0.3^c^	12±0.8^e^	26±0.2^a^	14±0.6^d^	20±0.4^b^

*S. aureus * ATCC 25923	25±0.0^a^	13±0.6^b^	24.9±0.3^a^	14.6±0.5^b^	25±0.8^a^

*S. epidemis * ATCC 12228	24±0.6^a^	20±0.3^b^	14±0.4^c^	13±0.0^c^	20±0.5^b^

*E. faecalis * ATCC 29212	18±0.1^b^	0^d^	20±0.3^a^	12±0.1^c^	12±0.2^c^

*M. luteus * ATCC 1880	21±0.4^a^	18±0.5^b^	15±0.6^c^	15±0.6^c^	20±0.7^a^

*L. monocytogenes* (food isolate 2132)	19±0.2^a^	18±0.1^a^	13.16 ±0.4^c^	12.60 ±0.9^c^	15±0.0^b^

**Gram** ^−^					

*S. enteritidis * (food isolate)	24±0.2^a^	14±0.3^c^	10±0.12^d^	11.3±0.1^d^	18±0.8^b^

*K. pneumoniae * ATCC 10031	18±0.2^a^	15±0.1^c^	16.6±0.3^b^	16±0.5^bc^	12±0.5^d^

**(b):** 

***Minimum inhibitory concentrations *(MIC) and* Minimum bactericidal concentration *(MBC) (mg/ml)**									

	**MIC**	**MBC**	**MIC**	**MBC**	**MIC**	**MBC**	**MIC**	**MBC**	

**Gram ** ^+^									

*B. subtilis* ATCC 6633	0.039	0.078	0.078	0.156	0.078	5.0	0.156	5.0	-

*B. cereus * ATCC 14579	0.039	0.625	0.156	5.0	0.156	0.156	0.312	5.0	-

*S. aureus * ATCC 25923	0.625	1.25	2.5	5.0	0.312	0.625	0.625	0.625	-

*S. epidemis * ATCC 12228	0.156	0.312	2.5	2.5	5.0	10	5.0	10	-

*E. faecalis * ATCC 29212	1.25	1.25	2.5	2.5	0.078	1.25	0.625	10	-

*M. luteus * ATCC 1880	0.312	1.25	2.5	5.0	5.0	10	5.0	10	-

*L. monocytogenes* (food isolate 2132)	0.156	0.625	2.5	5.0	1.25	5.0	1.25	5.0	-

**Gram** ^−^									

*S. enteritidis * (food isolate)	0.078	0.625	0.312	5.0	2.5	5.0	2.5	5.0	-

*K. pneumoniae * ATCC 10031	0.312	1.25	0.312	5.0	2.5	2.5	2.5	2.5	-

^*∗*^Genta: gentamicin was used as a standard antibiotic at a concentration of 15 *μ*g/well; essential oil concentration: 15 *μ*l/well; extract concentration: 2.5 mg/ml.

(-): not active (0 mm).

Values in the same line with different subscript (a > b > c>d) are significantly different within season (*P* <0.05).

## Data Availability

No data were used to support this study.
